# Prediction of clustered RNA-binding protein motif sites in the mammalian genome

**DOI:** 10.1093/nar/gkt421

**Published:** 2013-05-18

**Authors:** Chaolin Zhang, Kuang-Yung Lee, Maurice S. Swanson, Robert B. Darnell

**Affiliations:** ^1^Laboratory of Molecular Neuro-Oncology, Howard Hughes Medical Institute, The Rockefeller University, 1230 York Avenue, New York, NY 10021, USA, ^2^Department of Molecular Genetics and Microbiology and the Center for NeuroGenetics, University of Florida, College of Medicine, Gainesville, FL 32610, USA and ^3^Department of Neurology, Chang Gung Memorial Hospital, Keelung 204, Taiwan

## Abstract

Sequence-specific interactions of RNA-binding proteins (RBPs) with their target transcripts are essential for post-transcriptional gene expression regulation in mammals. However, accurate prediction of RBP motif sites has been difficult because many RBPs recognize short and degenerate sequences. Here we describe a hidden Markov model (HMM)-based algorithm mCarts to predict clustered functional RBP-binding sites by effectively integrating the number and spacing of individual motif sites, their accessibility in local RNA secondary structures and cross-species conservation. This algorithm learns and quantifies rules of these features, taking advantage of a large number of *in vivo* RBP-binding sites obtained from cross-linking and immunoprecipitation data. We applied this algorithm to study two representative RBP families, Nova and Mbnl, which regulate tissue-specific alternative splicing through interacting with clustered YCAY and YGCY elements, respectively, and predicted their binding sites in the mouse transcriptome. Despite the low information content in individual motif elements, our algorithm made specific predictions for successful experimental validation. Analysis of predicted sites also revealed cases of extensive and distal RBP-binding sites important for splicing regulation. This algorithm can be readily applied to other RBPs to infer their RNA-regulatory networks. The software is freely available at http://zhanglab.c2b2.columbia.edu/index.php/MCarts.

## INTRODUCTION

Mammals express hundreds of RNA-binding proteins (RBPs) interacting with specific target transcripts even in a single tissue like brain (1). Such interactions control multiple steps of RNA metabolism, including alternative RNA splicing and polyadenylation, mRNA export, editing, translation and turnover, contributing to specification of different cell types and developmental stages (2,3). Disruption of protein–RNA interactions resulting in misregulation of RNA is implicated in genetic diseases such as neurologic disorders and cancer (4,5).

Despite their central roles, inferring RNA-regulatory networks, especially on the global scale, has been impeded by both technical difficulties and the nature of protein–RNA interactions. Most RBPs recognize short (∼3–7 nucleotides or nt) and degenerate sequence motifs with limited information content. For example, the high-affinity binding sites of the prototypical neuron-specific splicing factor Nova are characterized by the tetramer YCAY (Y = C/U), as revealed by *in vitro* RNA selection (6,7), X-ray crystallography (8) and *in vitro* splicing assays (9,10). Other examples include recognition of YGCY sequences by Mbnl (11,12), UCUY by Ptbp1 (13) and Ptbp2 (14) and U-tracts by Hu (15,16) [reviewed by (17)]. Different strategies seem to have been used by RBPs to achieve sufficient targeting specificity (18), including co-expression of RBPs and their substrate transcripts in specific temporal or spatial windows to limit the search space, and cooperative binding of different RBPs to proximal sites (i.e. RNA motif modules) to stabilize each other. Another important mechanism is the additive or synergistic binding of multiple RNA-binding domains (RBDs) of an RBP (19). The latter appears to be a common scenario because many RBPs have multiple RBDs [e.g. 47% of annotated mouse RBPs (20)] and/or can multimerize, and thus several copies of the same sequence motif may be required for high-affinity and functional protein–RNA interactions. Following this notion, detailed biochemical and bioinformatic analysis demonstrated that three or more YCAYs clustered together are in general necessary and sufficient for Nova binding to RNA through the three KH domains, resulting in tissue-specific inclusion or exclusion of alternative exons (9,10,21,22).

Due to these complexities, few endogenous targets have been confidently identified for most RBPs until recently (17,23,24), which in turn has limited the capability of modeling RBP-binding specificity for effective and global prediction. Currently, RBP motifs are mainly represented by either sequence consensuses (25) or position weight matrices (PWMs) (26), which were originally used to describe transcription factor–binding motifs (27). However, prediction of individual motif sites using these models has limited discriminative power when applied to RBPs to determine target transcript-binding sites. This is further complicated by the fact that these RBP motifs were frequently derived from a small number of examples validated in separated studies, which may reveal biased representations of RBP-binding specificity. Therefore, with only a few exceptions (28), these motif models do not have enough specificity to predict novel RBP targets.

To map sites of protein–RNA interactions in an unbiased manner, we previously developed a biochemical assay named cross-linking and immunoprecipitation (CLIP) to isolate RNA fragments that are directly bound by an RBP (29,30). Combined with high-throughput sequencing (HITS-CLIP), this approach is able to map *in vivo* protein–RNA interactions at a genome-wide scale (30,31). Since then, several variations of HITS-CLIP were also developed by different groups (30). Importantly, a large number of high-quality RBP-binding sites identified in these experiments provide a training set so that richer probabilistic models can be applied to depict more sophisticated RBP-binding codes not apparent from a limited set of examples. Here we describe a hidden Markov model (HMM)-based algorithm and software tool, named mCarts (motif-based predictor of clustered accessible RBP target sites) that takes advantage of massive HITS-CLIP datasets to learn models of RBP-binding sites optimized for global prediction.

## MATERIALS AND METHODS

### The model of mCarts

**Figure 1.**
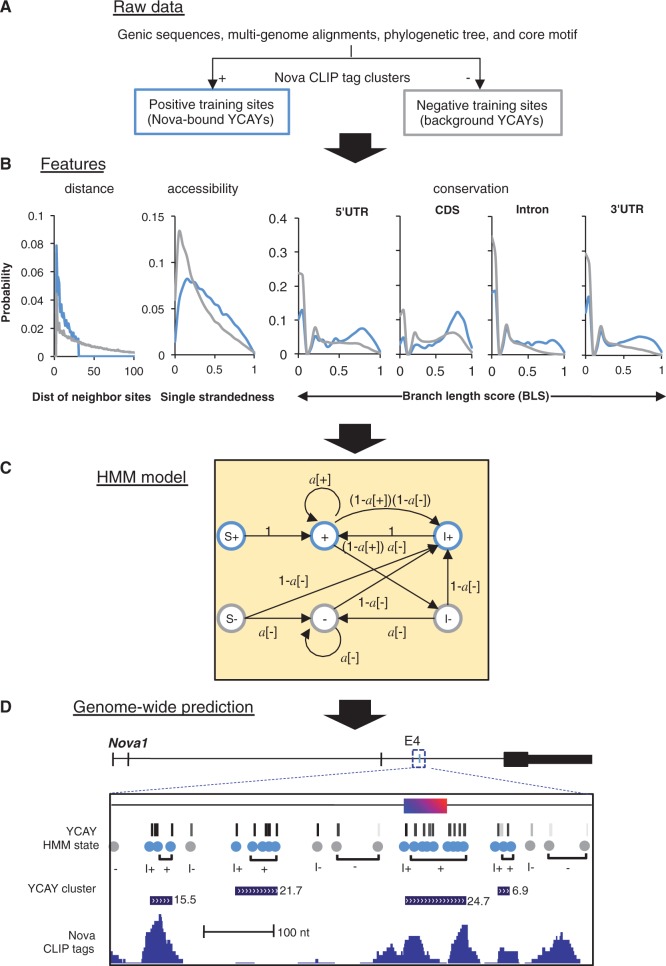
Overview of mCarts to predict clustered RBP motif sites using sequence, accessibility and conservation information. Prediction of Nova-bound YCAY clusters is used for illustration. (**A**) The proposed method uses motif sites in the CLIP tag clusters and sequences without CLIP tags as positive and negative training datasets, respectively. Motif sites are searched in these regions, and their distance to the preceding sites, accessibility and conservation are evaluated. (**B**) The distribution of each feature for sites in the positive (blue curves) or negative (gray curves) training dataset is estimated using a nonparametric representation. The distance between YCAYs in an RBP bound cluster (blue curve in the left panel) is censored at 30 nt, to impose an implicit limit of spacing allowed in a YCAY cluster. Conservation is modeled using BLS separately for different genomic regions. (**C**) The graphic representation of the HMM. Three states represent motif sites in an RBP-bound motif site cluster (blue), and the other three states represent motif sites in background sequences (gray). Detailed definition of each state, and their emission probability distribution, is summarized in (B) and Table 1. (**D**) The HMM model is used to predict RBP-bound motif site clusters in the whole transcriptome. The predicted clusters near *Nova1* exon4, a validated Nova target alternative exon, are shown as an example. In the zoom-in view, tracks shown are coordinates of YCAY elements with gray scale representing their conservation (BLS), the inferred HMM states, and predicted YCAY clusters and their scores, and Nova CLIP tags.

mCarts is motivated by the tandem arrangement of multiple RBDs in an RBP or the tendency of an RBP to multimerize, so that it can naturally characterize clusters of core motif sites by explicitly considering the variable spacing of individual sites, their accessibility and conservation across different species. In a previous effort, we developed an algorithm to predict clusters of YCAY elements (referred to herein as YCAY clusters) recognized by Nova, using several heuristic rules derived from analysis of a small number of Nova-regulated alternative exons (22). These rules considered clustering of YCAY elements and their conservation among eight vertebrate species. However, such a method is not optimized from a global perspective and cannot be readily applied to other RBPs. To overcome these limitations, we designed a general framework based on a HMM that can take advantage of CLIP data to optimize the model for specific RBPs and to predict clustered RBP-binding sites (Figure 1). This approach aims to improve the signal-to-noise ratio by integrating multiple types of information to score each individual motif site and also capture the clustering of multiple sites.

Specifically, the proposed method takes a set of sequences with robust CLIP tag clusters and background sequences (regions with no CLIP tags) as positive and negative training data, respectively, and compares motif sites in the two sets using features relevant to their biochemical or functional significance (Figure 1A and B, blue and gray in positive and negative datasets, respectively). The spacing (*d*) between neighboring motif sites is modeled explicitly as a feature given their importance for RBP binding. Motif conservation (*c*) is quantified using all mammalian or vertebrate species using branch length scores (BLS) (32), which were previously demonstrated to be effective in predicting binding sites of brain- and muscle-specific splicing factors of the RBFOX family (28). Due to the dramatic difference in basal conservation level, the distributions of conservation in 5′ and 3′ untranslated regions (UTRs), coding sequences (CDS) and introns are estimated separately. Moreover, we also model accessibility of motif sites (*a*), as represented by the probability of each site located in single-stranded regions, because *in vitro* selection (6,7), X-ray crystallographic data (8), CLIP data (24) and computational analysis (33,34) consistently suggested that RNA secondary structures can modulate the accessibility and function of RBP motif sites.

We designed a HMM consisting of six states to represent motif sites in an RBP-bound motif site cluster or background sequences (Figure 1C and Table 1). The first motif site in a RBP-bound motif site cluster is represented by S+ or I+, depending on whether it is the first site of an input sequence; the following sites in a RBP-bound cluster are represented by the state ‘+’. States to represent motif sites in background sequences (S-, I-, -) are defined similarly. Each individual motif site is an observation of one of the six states, characterized by 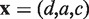 in the emission probability, while transition between states captures clustering of motif sites (Figure 1C). All possible transitions between states (directed edges in Figure 1C) are determined by the definition of each state (see the example in Figure 1D). All model parameters are estimated during training. During prediction, the state of each motif site is inferred by the Viterbi algorithm and clusters are defined and scored by the log-likelihood function log[P(**x**|cluster)/P(**x**|background)] (Figure 1D). Additional details of the algorithm are described in the Supplementary Notes.

**Table 1.** HMM emission probability distribution of each feature given the state

State (*s*)	Description	Pr(*d*|*s*)^a^	Pr(*c*|*s*), Pr(*a*|*s*)^a^
*S*+	Site in an RBP-bound motif cluster initiating an input sequence	Pr(0) = 1	+
*S*−	Site in a background region initiating an input sequence	Pr(0) = 1	−
+	Succeeding site in an RBP-bound motif cluster	+	+
−	Succeeding site in background sequences	−	−
*I*+	Site initiating an internal RBP-bound motif cluster	−	+
*I*−	Site initiating an internal background region	−	−

^a^‘+’ represents the distribution estimated from motif sites CLIP tag clusters (blue curves in Figure 1B); ‘−’ represents the distribution estimated from motif sites in background sequences (gray curves in Figure 1B).

### Software implementation

mCarts is currently implemented in Perl and C++, and is user friendly and flexible. The program takes a library of data files, which include multiple alignments of extended genic sequences, precalculated mRNA accessibility scores and gene structure annotations. In addition, the user provides the consensus motif sequence and the genomic regions to derive positive and negative training sites. The software, data library files (for mm9 and hg18) and documentation are freely available at http://zhanglab.c2b2.columbia.edu/index.php/MCarts.

### Compilation of CLIP data, exons, introns and alternative splicing events

HITS-CLIP data for Nova (24) and Mbnl2 (35) were described previously (SRA accessions: SRA019982 and SRA053472, respectively). Nova HITS-CLIP data are composed of >4.4 million unique tags, and the Mbnl2 CLIP data are composed of 703 431 unique tags, following filtering, genomic mapping and removal of polymerase chain reaction (PCR) duplicates as described in the original studies.

We searched motif sites of Nova and Mbnl in all genic sequences as defined by RefSeq and UCSC-known gene transcripts, with 10 000 nt extension on each side. These regions consisted of ∼1.45 G nt sequences in total, including 24 992 723 Nova high-affinity tetramer YCAYs and 15 205 158 Mbnl high-affinity tetramer YGCYs [with CGCC excluded (11)]. Annotations of exons, introns and alternative splicing events were generated by alignment of RefSeq, mRNA and EST sequences to the genome, as previously described (24). Repetitive regions were defined based on RepeatMasker (36), which was downloaded from the UCSC genome browser (37).

#### Model training and prediction

We did stringent filtering to get both positive and negative training data for the HMM. For Nova, the positive training dataset consisted of 6231 nonrepetitive genic CLIP tag clusters with peak height (PH) ≥ 15 and located in exons or 1 kb flanking intronic sequences on each side (exon + ext1k sequences). This threshold is roughly the median PH of CLIP tag clusters near Nova-regulated alternative exons. The negative training dataset consisted of 110 998 exon + ext1k sequences, in which no CLIP tags were present. The positive and negative sets included 34 058 and 2 124 463 YCAYs, respectively. For Mbnl, we similarly defined a positive training dataset consisted of 5536 nonrepetitive genic CLIP tag clusters with PH ≥ 7 in exon + ext1k regions, and a negative training dataset consisted of 171 357 exon + ext1k sequences without any CLIP tags. These datasets included 13 090 and 3 941 944 YGCYs (with CGCC excluded), respectively. The trained models were then used to predict clustered motif sites of Nova and Mbnl in all extended genic sequences, although only predicted clusters in genic sequences were presented in this article.

### Estimation of sensitivity and specificity using HITS-CLIP data

To test if inclusion of the training data in our transcriptome-wide prediction would introduce model overfitting and bias in performance evaluation, we performed a 2-fold cross-validation by splitting the whole transcriptome randomly into two halves. Two models were then trained using CLIP tag clusters and background sequences in one half, and tested on the other half, and *vice versa*. These models were denoted as cross-validation models. The predictions by the two cross-validation models on the independent halves were pooled together and compared with those predicted by the model trained on the full dataset. Specificity and sensitivity of prediction were evaluated by comparing Nova-binding YCAY clusters predicted by the cross-validation models with CLIP data using standard receiver operating characteristic (ROC) curves. We defined the region ±50 nt around the peak of a CLIP tag cluster as the ‘footprint’ region of Nova binding. The footprint regions of ∼2000 nonrepetitive robust CLIP tag clusters (PH ≥ 15) located in internal exons with 1 kb extension on each side were used as a surrogate of the true-positive dataset; to define a true negative set, we randomly sampled 100-nt sequences from exon + ext1k sequences without any CLIP tags. The reason we defined the footprint region for comparison was to eliminate the effect of variable sizes of different CLIP tag clusters, and the choice of particular size was empirical (based on observation of a limited number of validated Nova-binding sites). CLIP tag cluster footprints or background sequences were predicted as positive if an overlapping motif site cluster above a certain threshold was present, and negative otherwise, from which specificities and sensitivities were calculated. To evaluate the contribution of each individual feature, we built HMMs with various subsets of features (*d*+*c*, *d*+*a* and *d*) to make comparisons with each other and with the full model. All other model parameters were kept the same. ROC curves were calculated for each model.

### Predicting RBP-dependent splicing using clustered motif sites or HITS-CLIP data

We compared Nova or Mbnl target exons predicted by clustered motif sites or CLIP tag clusters with exons showing Nova- or Mbnl-dependent splicing as identified by independent splicing microarray or RNA-Seq data. Because multiple CLIP tag or motif site clusters typically exist in the alternatively splicing region, we derived a summarized CLIP tag or motif site cluster score by weighting each cluster according to their distance to the splice sites, as described previously (24). Each cassette exon was measured by six regional CLIP or motif site cluster scores denoted as *s*_UI5_′_ss_, *s*_UI3_′_ss_, *s*_E3_′_ss_, *s*_E5_′_ss_, *s*_DI5_′_ss_ and *s*_DI3_′_ss_ (UI: upstream intron; DI: downstream intron; E: exon). The maximum of them, denoted as summarized CLIP tag cluster score or summarized motif site cluster score, was used to rank exons. The thresholds used to predict Nova or Mbnl target exons shown in Figure 3 and Supplementary Figure S10 were determined somewhat arbitrarily. For comparison, we used a set of 483 cassette exons with Nova-dependent splicing, observed in at least one of four sets of Affymetrix exon or exon-junction microarrays that compared wild type (WT) and *Nova* knockout (KO) mice, as defined previously (24). Similarly, we used a set of 290 cassette exons with Mbnl2-dependent splicing, observed in exon-junction microarray or RNA-Seq data, as defined previously (35).

### Normalized complexity maps

The normalized complexity map of CLIP tags was generated as described previously (31,35). Briefly, we used a set of 325 nonredundant Nova target cassette exons confidently predicted by a Bayesian network approach or validated experimentally by reverse transcriptase–polymerase chain reaction RT-PCR (24), and 290 Mbnl2-dependent exons as determined by RNA-Seq or exon-junction microarrays (35). The normalized complexity map of clustered motif sites was generated similarly, except that motif site cluster scores in each alternatively spliced region were used to weight each cluster.

### RT-PCR validation of predicted Mbnl target exons

RNA extraction and RT-PCR for the analysis of Mbnl targets was performed as described previously (38). We used cDNA from quadriceps of WT and Mbnl1**^ΔE3/ΔE3^** (KO) mice as well as hippocampal cDNA from WT and Mbnl2**^ΔE2/ΔE2^** (KO) mice ranging from 3 to 6 months old, 129/BL6 mix-background mice. Each group included three biological replicates, and unpaired *t*-test was used for statistical analysis.

## RESULTS

### Prediction of Nova-binding YCAY clusters

As an application exemplifying the proposed method, we predicted Nova-bound YCAY clusters on a genome-wide scale. Nova proteins, encoded by two separate genes, *Nova1* and *Nova2*, are important for synaptic functions (39), in part through regulating alternative splicing as predicted by a position-dependent ‘RNA map’. In this map, alternative exons are included when Nova binds YCAY clusters in downstream introns and are excluded when Nova binds to YCAY clusters within the alternative exons or upstream introns (22,31). To define the comprehensive RNA-regulatory network of Nova, we recently generated in-depth mouse brain Nova HITS-CLIP data composed of >4.4 million unique tags (24). To train the HMM, we applied stringent filtering criteria to obtain matched sets of positive and negative training sequences from exons and 1 kilobase (kb) flanking intronic regions on each side (exon + ext1k sequences). All YCAY elements in these regions were extracted; spacing between neighboring elements, their accessibility and conservation were evaluated.

The large sample size of the training data made it possible to obtain precise nonparametric estimation on the distribution of each feature (Figure 1B and Supplementary Figure S1). All features showed clear differences between Nova-bound and background YCAYs, with smaller spacing, more accessibility and higher conservation for Nova-bound YCAYs. Therefore, all these features are expected to contribute to the prediction of Nova-bound YCAY clusters. Using this model, we predicted 841 501 potential Nova-bound YCAY clusters with ≥3 YCAYs in all genic regions of the mouse genome (Supplementary Dataset S1). Each cluster was assigned a log-likelihood score, denoted as YCAY cluster score, which is important to distinguish high-confidence predictions from those of low confidence (see below).

### Evaluation of predicted YCAY clusters using CLIP data

**Figure 2.**
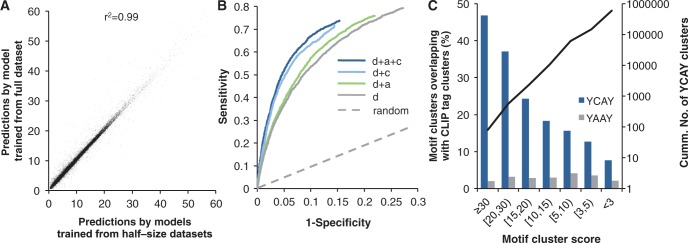
Evaluation of predicted YCAY clusters using CLIP data. (**A**) Correlation of YCAY cluster scores predicted by 2-fold cross-validation models (*x-*axis) and cluster scores predicted by the HMM trained on the full-size training dataset (*y-*axis). The squared Pearson correlation is indicated. (**B**) Comparison of cross-validation HMMs using all or subsets of features for the accuracy of Nova-bound YCAY cluster prediction. HMMs were trained on half-size training sets and evaluated on the independent test sets, as in *x-*axis in **(A)**. Specificity and sensitivity were estimated from the presence of predicted YCAY clusters in the footprint region of robust CLIP tag clusters (±50 nt of peaks, PH ≥ 15) or background sequences of the same size, and the resulting ROC curves are shown. Models using different subsets of features are compared: *d*, distance; *a*, accessibility; *c*, conservation. (**C**) The overlap between the footprints of CLIP tag clusters and predicted YCAY clusters with varying scores. Nonrepetitive YCAY clusters are binned into groups according to their scores. For each bin, the proportion of YCAY clusters overlapping with all CLIP tag cluster footprints (±50 nt of peaks) is shown (blue bars, left axis). YAAY clusters predicted by the same model are shown (gray bars) as a control. The cumulative number of nonrepetitive YCAY clusters is shown as the black curve (right axis).

We first evaluated the predicted YCAY clusters on a global scale by comparing them with CLIP data. Because CLIP tag clusters and background sequences used to estimate model parameters were also included in the prediction, we first performed 2-fold cross-validation to determine if the model is overfit. mCarts gave robust predictions, as reflected in both emission probabilities (Supplementary Figure S1) and the resulting YCAY cluster scores (Figure 2A) predicted by models derived from different training sets. Based on the presence of overlapping YCAY clusters, we were able to cross-validate the footprint regions of robust CLIP tag clusters and matched background sequences of the same size at a specificity of 85% and sensitivity of 74%, or a specificity of 99% and a sensitivity of 20% when we increased the threshold of the YCAY cluster score (≥7.3, and the median YCAY cluster score near validated Nova target exons is ∼15; Figure 2B). The latter represents a ∼20-fold increase in prediction specificity compared with random guesses. Prediction of YCAY clusters using models derived from subsets of features confirmed that all features used by mCarts indeed contributed to the accuracy of prediction (Figure 2B). Predictions based on spacing of YCAYs alone achieved the least satisfying performance, with 12% sensitivity at 99% specificity. Inclusion of conservation or accessibility together with spacing increased the sensitivity to 16.2% and 12.8% at the same specificity, respectively, which were nevertheless not as good as the full model (20%). Moreover, YCAY clusters with higher scores are more likely to overlap supporting CLIP tag clusters (Figure 2C), and more robust CLIP tag clusters with a larger PH are more likely to harbor predicted YCAY clusters (Supplementary Figure S2). In contrast, this was not observed for YAAY clusters predicted by the same model, which were not expected to be bound by Nova (9,10) and used as a control. This supports the notion that the YCAY cluster scores indeed reflect reliability of prediction quantitatively.

**Figure 3.**
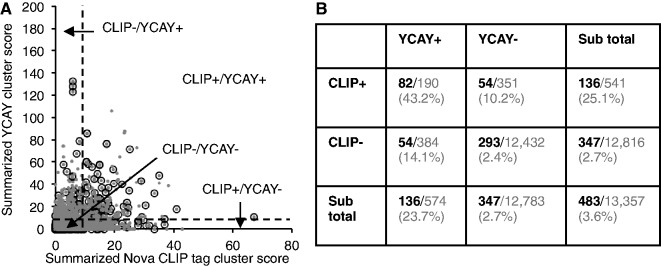
Nova-regulated alternative exons predicted from CLIP data and those predicted from YCAY clusters are complementary to each other. (**A**) Target exon scores predicted from CLIP data (*x-*axis) are plot against scores predicted from YCAY clusters (*y-*axis). Each gray dot is a cassette exon. All cassette exons are shown in gray, and exons with Nova-dependent splicing as determined by Affymetrix exon or exon-junction microarray data are overlaid in empty circles. A somewhat arbitrary threshold of summarized CLIP tag cluster score (10) or YCAY cluster score (10) is indicated by the dotted lines. (**B**) Breakdown of exons according to their summarized CLIP tag cluster score or YCAY cluster score above or below the threshold. The number (black and bold font) of exons currently with evidence of Nova-dependent splicing over the total (gray) in each category are also shown. The percentage is indicated in the parentheses.

### Extensive and distal YCAY clusters

**Figure 4.**
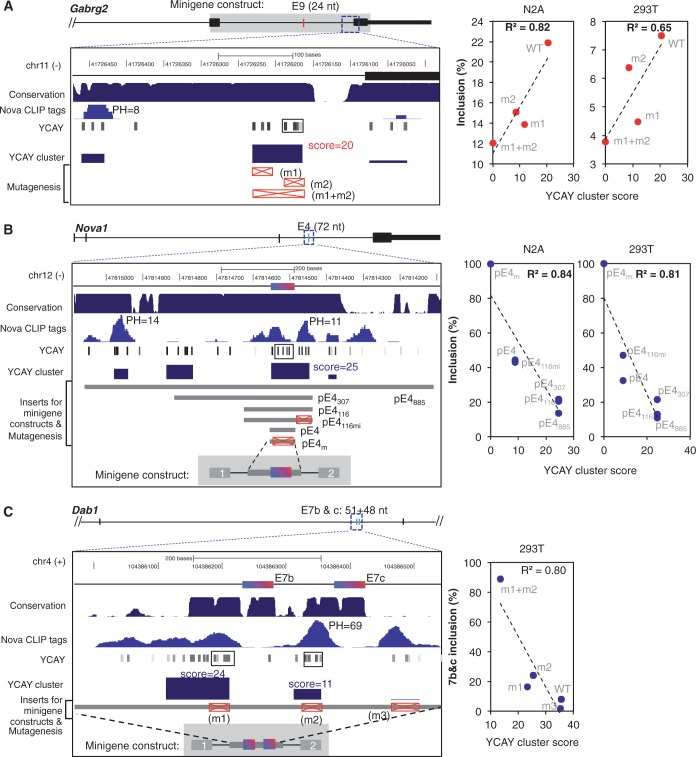
Mutagenesis validates predicted YCAY clusters. Mutagenesis analyses of Nova-binding YCAY clusters were previously performed in 293T or N2A cells for three splicing reporters. In each case, coordinates and schematic representation of the exon and intron structure, sequence conservation, CLIP tags and predicted YCAY clusters, as well as mutations introduced in the reporters are shown in the left panel. YCAY clusters predicted by our previous analysis (22) is indicated by a solid box in the YCAY track. The splicing of each reporter with WT or mutant YCAY clusters, in combination with transfection of Nova plasmids in N2A and/or 293T cells, was quantified by RT-PCR. Exon inclusion level of each reporter (*y-*axis) is correlated with the WT or mutant YCAY cluster score (*x-*axis), as shown on the right. The squared Pearson correlation coefficient is indicated. (**A**) *Gabrg2* exon 9 (10). The minigene consists of sequences between exons 8 and 10, as shaded in gray in the schematic representation of the gene structure. Mutant minigenes were generated by point mutations in the different sets of YCAY elements (YCAY→YAAY), as indicated by the red boxes with a cross. The analysis was performed in both N2A cells and 293T cells. (**B**) *Nova1* exon4 (9). The minigene constructs consist of *Nova1* exon 4 and flanking intronic sequences inserted into the human β-globin gene backbone. Mutant minigenes were generated by truncation of intronic sequences of different sizes covering the predicted YCAY clusters, together with point mutations in the YCAY elements (YCAY→YAAY), as indicated by the red boxes with a cross. The analysis was performed in both N2A cells and 293T cells. (**C**) *Dab1* exons 7b and c (41). The minigene constructs consist of exons 7b and c and flanking intronic sequences inserted into the human β-globin gene backbone. Mutant minigenes were generated by point mutations in different sets of YCAY elements (YCAY→YAAY), as indicated by the red boxes with cross. The analysis was performed in 293T cells. Inclusion of both exons 7b and c is shown.

The proposed algorithm is flexible in several aspects we believe to be important for modeling clustered RBP motif sites. First, it does not require arbitrary sliding windows, which would limit the number of individual sites in a cluster and the region a cluster can span (cluster width). Our previous analysis suggested that functional Nova-binding sites in general have ≥3 YCAY elements in a window of ∼45 nt (22), but these criteria likely represent the approximate minimal requirement. In fact, a number of YCAY clusters predicted by mCarts are far more extensive, with large variations in terms of the number of YCAY elements (Supplementary Figure S3A) and cluster width (Supplementary Figure S3B). In total, we predicted 3287 clusters with a score ≥15. These clusters typically consist of many YCAY elements in longer stretches of sequences. One such example is the YCAY cluster downstream of exon 6 in the *Ptprf* gene, which is strongly activated by Nova. (22). Strikingly, this YCAY cluster, scored 74 by mCarts, consists of 23 YCAY elements in a 200-nt region overlapping with highly conserved sequences and a reproducible CLIP tag cluster (PH = 11) (Supplementary Figure S4A). However, this exon received a moderate score (net YCAY score = 1.7) in our previous analysis (22), which was below the threshold (|net YCAY score| ≥ 2.7) previously used to predict Nova target exons, as a result of fragmentation of the YCAY cluster (see the black box in the YCAY track, Supplementary Figure S4A bottom panel; see Figure 4 below for more examples).

Second, mCarts has a natural representation of different types of sequences (i.e. CDS, untranslated regions and intron) with different baselines of conservation, which makes it possible to predict YCAY clusters that span exon–intron boundaries (e.g. Figure 1D), and also those distal from splice sites. Among the 2467 YCAY clusters with score ≥15 that are also ≥400 nt away from any annotated exons, a particularly interesting subset is 310 YCAY clusters distally located in flanking introns of cassette exons, including 135 clusters (43.5%) overlapping with CLIP tag clusters. One example is exon 17 of the *Ctnna2* gene, which was identified as a Nova-dependent exon by exon-junction microarrays (39). A strong YCAY cluster predicted by mCarts (score = 36), overlapping with a robust CLIP tag cluster (PH = 33), is located in the upstream intron, ∼1.6 kb downstream of the 5′ splice site; no other major YCAY clusters were predicted in the alternatively spliced region (Supplementary Figure S4B). The distance of this cluster to the nearest exon is much larger than that of typical splicing-regulatory elements. Another example of distal YCAY clusters is shown in Supplementary Figure S4C, and similar examples for other RBPs have also been reported (40), indicating that splicing regulation mediated by distal sites in introns might be a more general phenomenon.

### Prediction of Nova-regulated alternative exons using YCAY clusters

We next evaluated how YCAY clusters defined by mCarts can predict Nova-regulated alternative splicing. We first examined a set of 325 nonredundant Nova target cassette exons validated by RT-PCR or predicted by Bayesian networks (24). This analysis demonstrated that the position-dependent Nova RNA map based on predicted YCAY clusters, reflecting a population average of Nova action, was similar to the one derived from HITS-CLIP data (Supplementary Figure S5).

We then compared mCarts with our previous approach (22) in accuracy of predicting individual targets. Our previous method was first evaluated on a set of 48 Nova target cassette exons identified by CLIP (29), microarrays (39) and biochemical studies (9,10,21). To minimize the false-positive rate, a stringent threshold of ‘net YCAY score’ was previously determined so that 13 of the validated 48 exons (‘training set’) passed the threshold (sensitivity = 27.1%), which was then applied prospectively to predict 41 additional alternative exons in the mouse genome as new candidates (‘test set’). Among them, 20 exons were *bona fide* targets based on RT-PCR validation, giving a validation rate of 20/41 (48.8%). Using a threshold of mCarts YCAY cluster score = 21.1, we now predicted 15 of the 48 exons in the same training set (sensitivity = 31.3%), as well as 10 of 41 exons in the same test set, among which 9 are *bona fide* targets (validation rate = 90%) (Supplementary Figure S6). The only false-positive prediction of mCarts (*Sec24c,* YCAY cluster score = 28.7) overlapped with a strong CLIP tag cluster (PH = 21), although Nova-dependent splicing of this exon is currently not apparent. Therefore, YCAY clusters predicted by mCarts alone can identify a subset of top Nova target exons with a high validation rate, substantially outperforming our previous heuristic method.

Because our model was trained using CLIP data, an important question is whether the bioinformatic YCAY clusters provided additional information to determine functional Nova target exons. To address this question, we compared the performance of YCAY clusters and CLIP tag clusters in predicting Nova-dependent splicing, as measured independently by splicing microarrays that compared WT and *Nova* KO brains or spinal cords (24). When we predicted a similar number of Nova target exons using either summarized YCAY cluster scores (≥10) or CLIP tag cluster scores (≥10), respectively, a substantial proportion of exons (136/541 or 25.1% for CLIP data; 136/574 or 23.7% for YCAY clusters; 2.4% expected by chance; *P* < 10^−^^81^, Fisher′s exact test; Figure 3A and B) showed evidence of Nova-dependent splicing. Importantly, CLIP data and YCAY clusters predicted overlapping but distinct sets of exons. Among the exons predicted by both, a much higher proportion (82/190 or 43.2%; *P* < 10^−^^11^, Fisher’s exact test) had evidence of Nova-dependent splicing. Alternatively, if we consider the union of the targets predicted by CLIP or mCarts, we obtained ∼1.7-fold increase in the total number without sacrificing accuracy, compared with those predicted by CLIP data alone (925 versus 541). While microarray data might miss a substantial fraction of *bona fide* Nova target exons, these observations nevertheless suggest that YCAY clusters defined by mCarts and CLIP tag clusters in general had a comparable performance in predicting Nova target exons, and YCAY clusters provided additional information complementary to the CLIP data.

Separate studies in our lab have so far performed detailed mutagenesis analyses in three transcripts to define the exact Nova-binding sites important for alternative splicing regulation: GABA_A_ receptor γ2 (*Gabrg2*) exon 9 (10), *Nova1* exon 4 (9) and *Dab1* exons 7 b and c (41). In all these cases, the YCAY clusters independently predicted by mCarts precisely matched the experimentally validated Nova-binding sites (Figure 4A–C, left panel; the YCAY cluster with score indicated). These validated YCAY clusters are in general supported by CLIP tag clusters, although some exceptions were also observed. We note that some of the validated YCAY clusters were fragmented in our previous prediction (boxes in the YCAY track), presumably due to the use of sliding windows of a fixed size. We also evaluated the contribution of individual YCAYs to the affinity of Nova binding, and the impact of mutations on Nova-dependent alternative splicing. In all three examples, the predicted YCAY cluster scores in WT or mutant reporter sequences showed a strong correlation to the alternative exon inclusion level and explained a majority of variation (R^2^ between 0.80 and 0.84, except in one case R^2 ^= 0.65; Figure 4A–C, scatter plots on the right), again suggesting that the confidence of mCarts prediction reflects the functional significance of RBP motif sites.

### Prediction and evaluation of Mbnl-binding YGCY clusters

To assess whether mCarts can be generally applied to other RBPs, which also bind clusters of short and degenerate motif sites, we predicted Mbnl-binding sites on a genome-wide scale. In human, MBNL proteins, encoded by three members *MBNL1*, *MBNL2* and *MBNL3*, are key splicing factors in the neuromuscular disease myotonic dystrophy (DM) (4,42). A major molecular mechanism of DM is believed to be sequestration of the MBNL proteins by microsatellite C(C)UG expansions that contain their high-affinity YGCY elements (11,12), resulting in loss of MBNL function normally required to regulate alternative splicing of its endogenous target exons (43). This sequestration model was recently validated by splicing microarray analysis, which demonstrated similar global splicing defects in quadriceps muscles of mouse models either expressing a CUG repeat expansion (*HSA*^LR^) or depletion of Mbnl1 (*Mbnl1* KO) (12). While Mbnl1 is particularly important for splicing regulation in skeletal muscle, and Mbnl3 is expressed primarily in placenta (44), we recently demonstrated that Mbnl2 is expressed at a relatively high level in brain, including hippocampus, suggesting that this member of the Mbnl family is particularly important for CNS function (35). Indeed, using exon-junction microarrays and RNA-Seq on hippocampal tissue, we found hundreds of alternative exons whose splicing was altered on Mbnl2 depletion in mice, suggesting that Mbnl2 loss-of-function might explain several CNS phenotypes, including hypersomnia and learning/memory deficits observed in both *Mbnl2* KO mice and DM patients (35). Mbnl2 HITS-CLIP experiments showed that Mbnl2 binds to tetramers that follow the YGCY consensus (except CGCC), which is similar to the specificity of Mbnl1 (11,12). Correlating CLIP data with microarray and RNA-Seq results suggested that Mbnl2 regulates alternative splicing by interacting with sequences enriched in YGCY elements, whose positions determine exon inclusion or exclusion, in a manner analogous to Nova.

We focused initially on Mbnl2 because of the availability of HITS-CLIP data. However, we expect that Mbnl2-binding sites may have a substantial overlap with those of the other two members of the family owing to the similarity in their motifs. Following the same pipeline used to analyze Nova, we defined matched sets of positive and negative training data based on Mbnl2 CLIP data, and searched all YGCY elements (CGCC excluded, the same below) therein. In general, YGCY elements in CLIP tag clusters are more clustered, and have higher cross-species conservation (Supplementary Figure S7), qualitatively similar to what we observed with Nova. However, we did not observe a clear preference for Mbnl to recognize YGCYs in single-stranded sequences, in contrast to Nova, which is consistent with the sequestration of MBNL by the pathogenic hairpin structure formed by CUG repeats in DM (4,42).

mCarts predicted 277 632 potential Mbnl-binding YGCY clusters with ≥3 YGCYs in all genic regions, which were ranked according to the log likelihood score or YGCY cluster score (Supplementary Dataset S2). When we overlaid the predicted YGCY clusters with CLIP tag clusters, YGCY clusters with higher scores showed a higher overlap, and *vice versa* (Supplementary Figure S8), again suggesting that the YGCY cluster score can be used to separate high-confidence predictions from those of low confidence. In contrast, control YACY clusters predicted by the same model, which are presumably not bound by Mbnl due to lack of the core GC dinucleotide (11), had a much lower overlap with CLIP data, and the magnitude of overlap did not depend on the stringency of YACY clusters or CLIP tag clusters.

### Prediction and validation of Mbnl-regulated alternative exons using YGCY clusters

We next correlated predicted YGCY clusters with exons showing Mbnl-dependent splicing. Overall, YGCY clusters were enriched near 5′ and 3′ splice sites in the downstream introns for exons showing Mbnl2-dependent inclusion, and in the upstream introns for exons showing Mbnl2-dependent skipping. This matched well the patterns we observed from CLIP data (Supplementary Figure S9).

The predicted YGCY clusters also provided sufficient specificity to determine individual Mbnl-regulated exons. By requiring a summarized YGCY cluster score ≥10 (see ‘Materials and Methods’ section for more details), we predicted 392 cassette exons from 263 genes to be regulated by Mbnl. A significant fraction (48/392 or 12.2%) of these exons showed evidence of Mbnl2-dependent splicing as observed in exon-junction microarrays or RNA-Seq data (35), while only 1.9% is expected by chance (*P* = 3.7 × 10^−^^21^, Fisher’s exact test). These genes showed enrichment of specific gene ontology terms (45), such as small GTPase binding (false discovery rate or FDR < 7.3 × 10^−^^3^), synapse (FDR < 2.0 × 10^−^^3^) and cell projection (FDR < 1.5 × 10^−^^2^), as compared with all genes containing cassette exons, consistent with the role of Mbnl in neuromuscular function. The concordance of exons predicted by YGCY clusters with exons showing Mbnl2-dependent splicing is also comparable with that of CLIP data, if we predicted a similar number of exons by summarized CLIP tag cluster score ≥3.8 (49/395 or 12.4%) (Supplementary Figure S10). Importantly, exons predicted by both YGCY clusters and CLIP data showed higher concordance with Mbnl2-dependent splicing (19/70 or 27.1%) than those predicted by either CLIP or YGCY clusters alone (*P* < 0.003, Fisher’s exact test). Again, if we consider the union of the targets predicted by CLIP or mCarts, we obtained ∼1.8-fold increase in total number without sacrificing accuracy, compared with those predicted by CLIP data alone (717 versus 395). This supports the notion that YGCY clusters also provided additional information complementary to the CLIP data.

**Figure 5.**
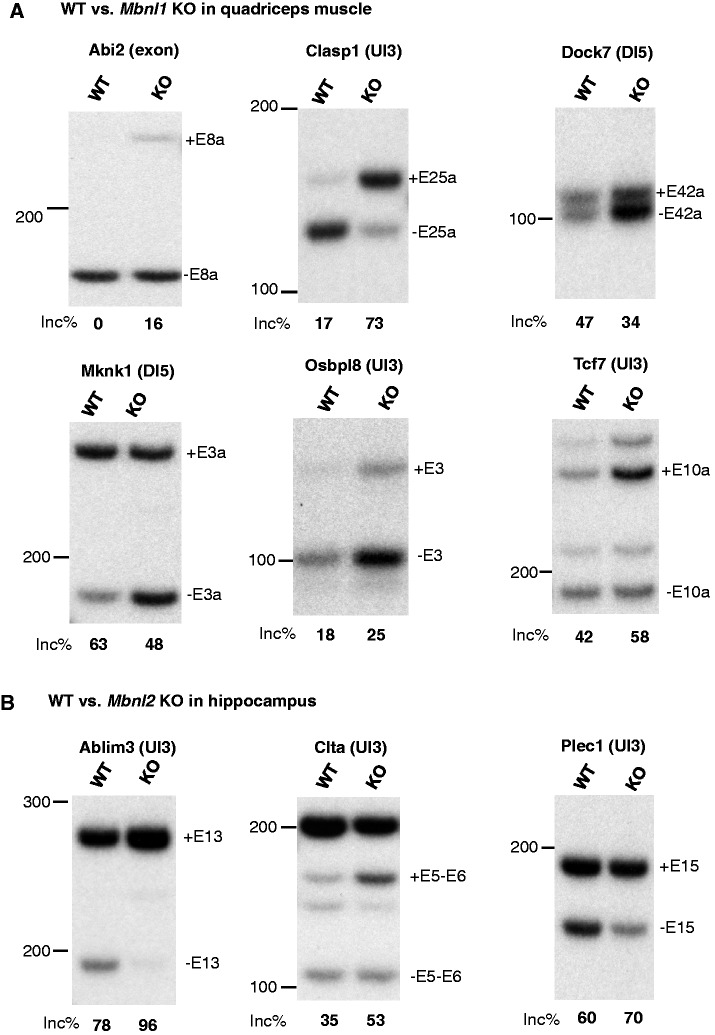
Semi-quantitative RT-PCR validation of predicted Mbnl target alternative exons. (**A**) Six exons showing Mbnl1-dependent exon inclusion or exclusion in comparison of WT and *Mbnl1* KO quadriceps muscles. (**B**) Three exons showing Mbnl2-dependent exon inclusion or exclusion in comparison of WT and *Mbnl2* KO hippocampus. For each exon, three biological replicates of WT and three biological replicates of *Mbnl1* or *Mbnl2* KO samples were used. The typical gel image is shown with the average percent exon inclusion indicated below. The band representing the inclusion or skipping isoform is labeled on the right, with the sizes of molecular markers indicated on the left. The position of the major YGCY clusters predicted by mCarts is indicated in the parentheses following the gene symbol (UI3: 3′ end of the upstream intron, DI5: 5′ end of the downstream intron). In all cases, the splicing changes on Mbnl1 or Mbnl2 depletion are statistically significant (*P* < 0.05; *t*-test).

**Figure 6.**
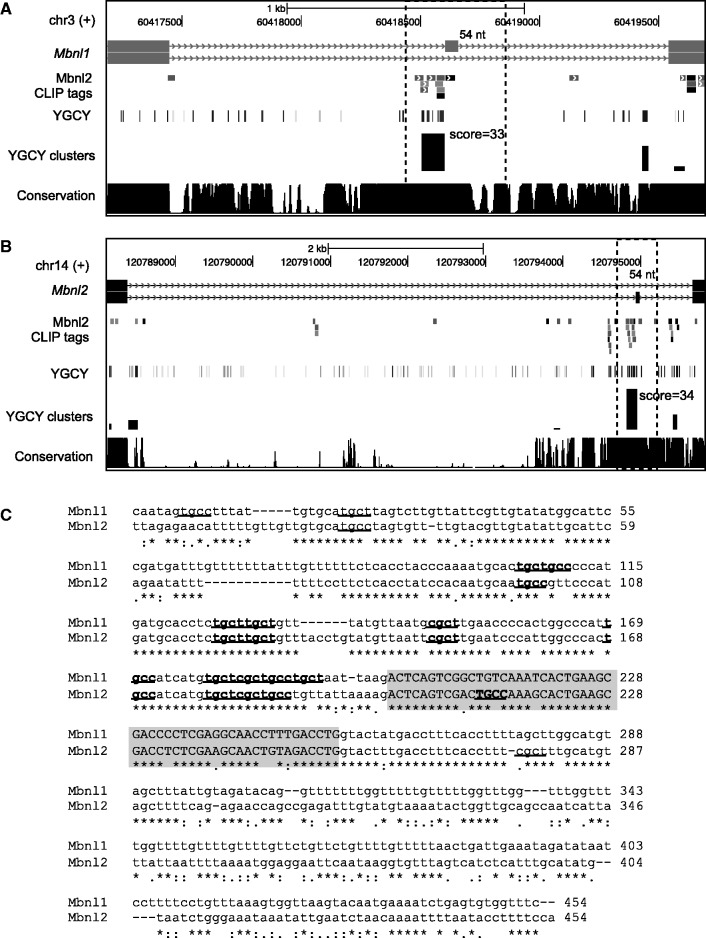
Mbnl1 and Mbnl2 are autoregulated through alternative splicing. (**A**, **B**) Both Mbnl1 (A) and Mbnl2 (B) have a 54 nt alternative exon, which showed Mbnl-dependent splicing. In both cases, a strong YGCY cluster was predicted in the upstream intron near the 3′ splice site, where robust CLIP tags were mapped. (**C**) Alignment of the alternative exon (shaded) and flanking intronic sequences in Mbnl1 and Mbnl2 (dotted boxes in A and B) are shown. YGCY elements are highlighted by underscores, and those in predicted YGCY clusters are shown in bold.

We then focused on the top 30 nonoverlapping exons predicted by YGCY clusters (summarized YGCY cluster score ≥20.2) for detailed analysis of splicing regulation by Mbnl2 in hippocampus and Mbnl1 in quadriceps muscle (Supplementary Table S1). Among these, 8 exons previously showed Mbnl2-dependent splicing in hippocampus (35), and 3 additional exons were previously validated to be regulated by Mbnl1 in quadriceps muscle (12) (11/30 or 36.7%). The inclusion of both Mbnl1 and Mbnl2 targets in predictions is expected, because they both recognize YGCY elements. Because the RNA-Seq or exon-junction microarray data used in previous studies likely missed a number of *bona fide* Mbnl targets, owing to their relatively moderate statistical power, we tested predicted exons currently without evidence of Mbnl1/2-dependent splicing by performing semi-quantitative RT-PCR, using WT or *Mbnl1*- or *Mbnl2*-KO mice. Among the 12 exons tested, we were able to draw conclusions on 11 exons, including 9 exons (81.8%) that showed Mbnl1-dependent splicing in muscle or Mbnl2-dependent splicing in hippocampus, and 2 exons that showed no changes on Mbnl1 or Mbnl2 depletion (Figure 5A and B, Supplementary Tables S1 and S2). Therefore, the overall validation rate of the top 30 predictions is 88.5% (0.367 + 0.633 × 0.818). Note that a majority of these exons (22/30) have few or no CLIP tags (summarized CLIP tag cluster score <3.8) in the alternatively spliced region, probably due to the relatively limited depth of the CLIP data. In all cases, with only one exception (*Camk2g*), downstream YGCY clusters correctly predicted Mbnl-dependent exon inclusion, while upstream or exonic YGCY clusters correctly predicted Mbnl-dependent exclusion. For *Camk2g*, the predicted YGCY cluster is in the downstream intron, but only 8 nt from the 5′ splice site, which will potentially block the access of the 5′ splice site to the spliceosome, and therefore explain its repressive effect. Intriguingly, a 54-nt homologous cassette exon in both *Mbnl1* and *Mbnl2*, which is developmentally regulated to affect the nuclear localization of Mbnl proteins (46), is predicted among the top candidates by YGCY clusters. In both cases, this exon showed Mbnl2-dependent exclusion in hippocampus in RNA-Seq data, and the Mbnl1 exon was also tested and validated by RT-PCR (35). The high-scoring YGCY cluster (score = 33 and 34, respectively) is located upstream of the alternative exon, and supported by overlapping CLIP tag clusters (Figure 6). Therefore, like many other splicing factors, Mbnl autoregulates its own expression at the splicing level.

## DISCUSSION

Here we present mCarts, a computational method capable of predicting clustered RBP motif sites on the genome-wide scale. This method combines several features intrinsically or extrinsically important for specific binding of RBPs to their target transcripts and function of such interactions. Predictions of Nova-binding YCAY clusters using this algorithm have been integrated with additional information including CLIP data, Nova-dependent splicing detected in microarrays, as well as evolutionary signatures, to define the Nova alternative splicing target network (24). Here we describe the method and make the software available for the community. We systematically evaluated its performance to show its reliability and complementarity with the biochemical CLIP data, and discovery of novel functional RBP motif sites that are unexpectedly extensive and distal. As an important extension, we also demonstrate its general applicability by applying mCarts to study another representative splicing factor Mbnl to predict its binding sites and target exons, and show that predicted novel target transcripts can be successfully validated.

Mapping *in vivo* protein–RNA interactions has been challenging until recently. Splicing-sensitive microarrays (39) or more recently RNA-Seq (35) in combination with perturbation of specific RBPs do not distinguish direct or indirect targets, or pinpoint the exact binding sites. *In vitro* selection (6) to purify short RNA oligos with high affinity to specific RBPs do not reflect their function *in vivo*. Mutation analysis was able to identify exact sites of functional significance, but was labor intensive (9,10,41). Applications of high-throughput biochemical assays including HITS-CLIP and its variants (30) provide a means of mapping *in vivo* protein–RNA interactions on a global scale, which are critical to train any data-driven probabilistic models, including mCarts proposed in this study.

However, bioinformatic prediction of RBP motif sites is complementary to biochemical protein–RNA interaction footprints in several regards. First, one major goal of investigating RNA-regulatory networks is to understand how the genetic information coded in the genome, including the position and strength of *cis*-regulatory elements, can be unfolded to orchestrate gene expression. Modeling and characterization of RBP-binding sites at the sequence level provide a more mechanistic, rather than empirical, view of protein–RNA interactions, which will eventually be essential to interpret sequence variations and mutations in evolutionary, population and disease studies. In addition, the ability of bioinformatic predictions to determine exact motif sites is also complementary to the ∼30–60 nt resolution of CLIP data and important for certain applications such as mutagenesis and therapeutic interference, although this is addressed in part by cross-linking induced mutation site analysis (47).

Second, the comprehensiveness of CLIP data largely depends on the complexity of the library and the depth of sequencing, especially for transcripts with low abundance, restrictive expression in specific cell types or fast degradation. CLIP might also miss some sites owing to several technical issues, such as inaccessibility of certain sequences [e.g. RNase A cuts only single-stranded sequences, and RT may stall at some cross-link sites (47)]. In line with these arguments, some Nova target exons have predicted YCAY clusters validated to be critical, but few supporting CLIP tags were observed (e.g. *Gabrg2* exon 9 in Figure 4A, *Glra2* exons 3a and b described in (6,48), and additional examples in Supplementary Figure S6). Therefore, the true-positive rate of predicted RBP motif sites is likely higher than that estimated from comparison with CLIP data (Figure 2B and C, and Supplementary Figure S8).

Third, the biochemical protein–RNA interactions detected by CLIP is a snapshot of the specific condition under investigation, and CLIP tag cluster peak height represents a composite measurement of binding affinity and transcript abundance. On the other hand, bioinformatic predictions of RBP motif sites are independent of transcript abundance and not limited to genes expressed under specific conditions. Some predicted motif sites without support from CLIP could be *bona fide* binding sites of the RBP in other conditions. However, we also expect bioinformatic methods may include false positive predictions in sequences that RBPs never bind, as the current model almost certainly does not capture all the mechanistic details of protein–RNA interactions. Ranking of predicted RBP motif sites by confidence scores helped to reduce false positives, and the high-scoring sites favor those with high cross-species conservation (Supplementary Figure S4B and C). It is particularly encouraging that among the top candidate exons predicted to be direct RBP targets, a high validation rate was achieved (90% and 88.5% for Nova and Mbnl, respectively).

mCarts extended several previous efforts that attempted to predict *cis*-regulatory elements in RNA, and some of them predicted splicing-regulatory elements (6–8 mer words) enriched in exons or introns without restricting analysis to specific RBPs (25). It is not surprising that these regulatory elements (more precisely, motifs) do not provide sufficient specificity to predict individual target transcripts, which was reflected in varying effects depending on context sequences when they were inserted into different reporters (49,50). The binding motifs of specific RBPs were also determined experimentally by *in vitro* selection or other approaches, and the resulting short consensus sequences or PWMs were used to search exonic or intronic sequences to predict putative splicing-regulatory elements. These predictions similarly suffered from the low information content of the motif representation. For example, when a Ptbp1-binding consensus sequence (YYYYUCUUYYYY) was used for genome-wide search (51), only 1% of predicted sites overlapped with CLIP tag clusters (52).

To overcome the small size and high degeneracy of RBP motifs, we previously derived a set of heuristic rules specifically tailored to predict YCAY clusters recognized by Nova (22). However, such a method was neither optimized from a global perspective due in part to the limited size of the training set, nor can it be readily adapted to study other RBPs. A more general method named SFmap was proposed more recently (53). This method weights multiple motif sites in a sliding window based on their similarity with the consensus and pairwise conservation in human and mouse. However, both scoring functions and the size of sliding windows were chosen in a somewhat *ad hoc* manner independent of specific RBPs. The use of pairwise conservation also limited the discriminative power comparative analysis of many sequenced species can provide to identify sequences under strong selection.

Compared with these previous efforts, mCarts provides the generalizability that can be readily applied to different RBPs, and also the capability to be optimized for specific RBPs in a data-driven manner, with minimal prior knowledge and assumptions. Variations of HMMs have been applied to model combinations of different transcription factor–binding motifs (54–57) and miRNA target sites (58). Our model is specifically designed to predict protein–RNA interactions according to several distinct features, including spacing of individual sites, their accessibility and conservation. As we described above, combination of these features in a unified framework greatly improved the accuracy of prediction, partly by more quantitative modeling of these features and elimination of strict limits on cluster size, a caveat in previous approaches (22,53). The effectiveness and general applicability of the method were demonstrated in its application on two representative RBP families Nova and Mbnl, resulting predicted motif sites that have substantial concordance with CLIP data. It also predicted alternative exons regulated by each protein, as evaluated by independent splicing microarray or RNA-Seq data and RT-PCR validation, and many of these are complementary to those predicted from CLIP data. Given that an increasing amount of experimental data to determine binding sites of various RBPs is being generated using CLIP (30) and other technologies, we expect that the proposed method and the software tool has the potential to facilitate the characterization of protein–RNA interactions and the construction of RNA-regulatory networks.

## SUPPLEMENTARY DATA

Supplementary Data are available at NAR Online: Supplementary Tables 1–2, Supplementary Figures 1–10, Supplementary Notes, Supplementary Datasets 1–2 and Supplementary References [6–8,22,24,26,32–34,53,59–61].

## FUNDING

National Institutes of Health (NIH) [NS34389 to R.B.D., NS058901 to M.S.S. and K99GM95713 to C.Z.]; the Rockefeller University Hospital CTSA [UL1 RR024143 to R.B.D.]. R.B.D. is an HHMI Investigator. Funding for open access charge: NIH [NS34389 to R.B.D.].

*Conflict of interest statement*. None declared.

## Supplementary Material

Supplementary Data
