# Supplementary material for: Prediction of clustered RNA-binding protein motif sites in the mammalian genome
Source: Nucleic Acids Res. 2013 May 18;41(14):6793–807. doi: 10.1093/nar/gkt421 (PMC3737533; doi:10.1093/nar/gkt421)
Supplement: Supplementary Data [file supp_41_14_6793__index.html]

Prediction of clustered RNA-binding protein motif sites in the mammalian genome — Prediction of clustered RNA-binding protein motif sites in the mammalian genome — Supplementary Data 

# Prediction of clustered RNA-binding protein motif sites in the mammalian genome

## Supplementary Data

files

**Files in this Data Supplement:**

- Supplementary Data - pdf file
