# Supplementary material for: Prediction of clustered RNA-binding protein motif sites in the mammalian genome
Source: Nucleic Acids Res. 2013 May 18;41(14):6793–807. doi: 10.1093/nar/gkt421 (PMC3737533; doi:10.1093/nar/gkt421)
Supplement: Supplementary Data [file supp_gkt421_nar-00859-z-2013-File008.pdf]

Supplementary Information

Prediction of clustered RNA-binding protein motif sites in the mammalian genome

Chaolin Zhang,[1,4,*] Kuang-Yung Lee[2,3], Maurice S. Swanson[2], Robert B. Darnell[1,*]

[1] Laboratory of Molecular Neuro-Oncology, Howard Hughes Medical Institute, The Rockefeller University, 1230 York Avenue, New York, NY 10021, USA

[2] Department of Molecular Genetics and Microbiology and the Center for NeuroGenetics, University of Florida, College of Medicine, Gainesville, FL 32610, USA

[3] Department of Neurology, Chang Gung Memorial Hospital, Keelung 204, Taiwan

[4] Present address: Department of Biochemistry and Molecular Biophysics, Columbia Initiative in Systems Biology, Center for Motor Neuron Biology and Disease, Columbia University, New York NY 10032, USA

[*] To whom correspondence should be addressed:
Chaolin Zhang
Department of Biochemistry and Molecular Biophysics
Columbia Initiative in Systems Biology
Center for Motor Neuron Biology and Disease
Columbia University
New York, NY, 10032
Tel: (212)305-9354
Fax: (212)342-4512
Email: cz2294@columbia.edu

Robert B. Darnell
Howard Hughes Medical Institute
Laboratory of Molecular Neuro-Oncology
The Rockefeller University
New York, NY, 10065, USA
Tel: (212) 237-7460
Fax: (212) 327-7109
Email: darnelr@rockefeller.edu

Supplementary Notes: Specification of the HMM

Emission probability

The HMM underlying mCarts consists of six states, among which three states represent RBP-bound motif sites at different positions of a cluster (S+, I+, +, shown in blue in Fig. 1C) and three other states represent background motif sites (S-, I-, -, shown in gray, Fig. 1C). Each motif site (e.g., YCAY element) is an observation of one of the six states characterized by three types of features in the emission probability: clustering of neighboring sites (d), their accessibility (a) and conservation (c), as summarized in Table 1.

Clustering of motif sites is explicitly modeled by the distance of each site to the preceding site (denoted as d), except the very first site in an input sequence (states S+ and S-, Fig. 1C and Table 1), for which a dummy distribution was used (Table 1). The distance of the second and additional succeeding sites in a cluster (state +) followed the same distribution estimated from motif sites in CLIP tag clusters ($P(d|+)$, e.g., blue curve in the left panel, Fig. 1B), while the distance of any other states followed another distribution estimated from YCAYs in background sequences ($P(d|s)$, s={I+, I-, -}, gray curve in the left panel, e.g., Fig. 1B). We also censored the distribution for motif sites belonging to a RBP-bound cluster [$P(d|+)$, e.g., blue curve in the left panel, Fig. 1B], which imposed an implicit limit on the maximum spacing allowed for individual motif sites in an RBP-bound cluster. For this study, we required $d \leq 30$, a threshold determined empirically by examining validated Nova and Mbnl target exons (see below), which is much less restrictive than several previous studies (1,2). However, we also tried other thresholds (e.g., 20 and 50) and obtained qualitatively similar results.

Motif conservation (denoted as c) was quantified using multiple alignments of 20 mammalian species (3) by branch length scores (BLS) (4), which were previously demonstrated to be effective in predicting binding sites of brain- and muscle-specific splicing factors of the RBFOX family (5). Because the basal conservation level of sequences in different genomic regions varies dramatically, we modeled motif site conservation separately for sites in 5′ untranslated regions (UTRs), coding sequences (CDS), introns and 3′ UTRs.

In vitro selection (6,7), X-ray crystallographic data (8), CLIP data (9) and computational analysis (10,11) consistently suggested that RNA secondary structures can modulate the accessibility and function of RBP motif sites. The accessibility of a motif site (denoted as a) in local RNA secondary structures was measured by its probability of being located in single-stranded region, as predicted by the RNAplfold program (parameters: -u 4 for tetramer motif, and a default window size of 70 nt) in the ViennaRNA package (12). The probabilistic distributions of these features were estimated nonparametrically from motif sites in training CLIP tag clusters and background sequences, respectively, and represented by histograms (e.g., Fig. 1A and B). Since conservation and accessibility scores are continuous, these variables were discretized into a specified number of bins (e.g., 20 bins in this study).

Together, each motif site can be denoted by $\mathbf{x}_{i,l_i} = (d_i, a_i, c_i)$ (i=1,2,…, N), in which i is the index of the motif site, and l_i is an indicator that records the genomic location of the site (5′ or 3′ UTRs, CDS, or introns). For simplicity, these features are assumed to be independent with each other to estimate the overall emission probability:

$$e(s_i, \mathbf{x}_{i,l_i}) = P(\mathbf{x}_{i,l_i} \mid s_i) = P(d_i \mid s_i)P(c_i \mid s_i, l_i)P(a_i \mid s_i), \quad (1)$$

where s_i represents the state of the site.

Transition between states

Legitimate transitions between states $t(s_i, s_{i+1})$ are naturally determined by the definition of a motif cluster while the other transitions are not allowed to occur (Fig. 1 C). We denote the probability of staying at the "+" state and the "-" as a[+] and a[-], respectively, and they can be estimated from the average duration (number) of sites in each CLIP tag cluster (μ[+]) and background sequences (μ[-]), respectively, a[+]=1-1/ μ[+], and a[-]=1/ μ[-]. Since the size of each background sequence was chosen arbitrarily, for this study we estimated μ[-] based on the relative frequency of YCAYs in positive versus negative training set r, μ[-] = μ[+]/r. Transitions between different states can be calculated accordingly, as summarized in Fig. 1C.

Model training and prediction

The parameters of the model $\lambda = (t, e)$ (transition probabilities and emission probabilistic distributions) were estimated from the training data. During prediction, each input sequence (i.e., whole transcripts including introns with 10 kb extension on both sides for this study), which usually contains a sufficiently large number of motif sites, is processed independently. The model takes the motif sites (represented by features described above) in each sequence as input, and decodes the state of each site by the Viterbi algorithm to maximize the joint likelihood of the hidden states and the observed variables (13).

$$\begin{aligned} &P(s_1, s_2, ..., s_N, \mathbf{x}_{1,l_1}, \mathbf{x}_{2,l_2}, ..., \mathbf{x}_{N,l_N} \mid \lambda) \\ &= P(s_1, s_2, ..., s_N \mid \lambda) P(\mathbf{x}_{1,l_1}, \mathbf{x}_{2,l_2}, ..., \mathbf{x}_{N,l_N} \mid s_1, s_2, ..., s_N, \lambda) \\ &= P(s_1) \prod\nolimits_2^N P(s_i \mid s_{i-1}) \prod\nolimits_1^n P(\mathbf{x}_{i,l_i} \mid s_i) \end{aligned} \quad (2)$$

RBP-bound clusters are defined to be a consecutive series of states S+, +, …,+, or I+, +, …,+, and ranked by the log-likelihood ratio:

$$C = \log\left\{\frac{P(\mathbf{x}_{1,l_1}, \mathbf{x}_{2,l_2}, \ldots, \mathbf{x}_{n,l_n} \mid s_1 = I+, s_2 = +, \ldots, s_n = +)}{P(\mathbf{x}_{1,l_1}, \mathbf{x}_{2,l_2}, \ldots, \mathbf{x}_{n,l_n} \mid s_1 = I-, s_2 = -, \ldots, s_n = -)}\right\}$$

$$= \log\left\{P(\mathbf{x}_{1,l_1} \mid s_1 = I+) / P(\mathbf{x}_{1,l_1} \mid s_1 = I-)\right\} \qquad , \qquad (3a)$$

$$+\sum_{i=2}^{n} \log\left\{P(\mathbf{x}_{i,l_i} \mid s_i = +) / P(\mathbf{x}_{i,l_i} \mid s_i = -)\right\}$$

for a cluster in which the first site is the very first site in the input sequence, or otherwise

$$C = \log\left\{\frac{P(\mathbf{x}_{k,l_k}, \mathbf{x}_{k+1,l_{k+1}}, \ldots, \mathbf{x}_{k+n-1,l_{k+n-1}} \mid s_k = I+, s_{k+1} = +, \ldots, s_{k+n-1} = +)}{P(\mathbf{x}_{k,l_k}, \mathbf{x}_{k+1,l_{k+1}}, \ldots, \mathbf{x}_{k+n-1,l_{k+n-1}} \mid s_k = I-, s_{k+1} = -, \ldots, s_{k+n-1} = -)}\right\}$$

$$= \log\left\{P(\mathbf{x}_{k,l_k} \mid s_k = I+) / P(\mathbf{x}_{k,l_k} \mid s_k = I-)\right\} \qquad , \qquad (3b)$$

$$+\sum_{i=k+1}^{k+n-1} \log\left\{P(\mathbf{x}_{i,l_i} \mid s_i = +) / P(\mathbf{x}_{i,l_i} \mid s_i = -)\right\}$$

where k (>1) is the first motif site in a cluster and n is number of motif sites of the cluster.

Supplementary references

1. Akerman, M., David-Eden, H., Pinter, R. and Mandel-Gutfreund, Y. (2009) A computational approach for genome-wide mapping of splicing factor binding sites. *Genome Biol.*, **10**, R30.
2. Ule, J., Stefani, G., Mele, A., Ruggiu, M., Wang, X., Taneri, B., Gaasterland, T., Blencowe, B.J. and Darnell, R.B. (2006) An RNA map predicting Nova-dependent splicing regulation. *Nature*, **444**, 580-586.
3. Pollard, K.S., Hubisz, M.J., Rosenbloom, K.R. and Siepel, A. (2010) Detection of nonneutral substitution rates on mammalian phylogenies. *Genome Res.*, **20**, 110-121.
4. Stark, A., Lin, M.F., Kheradpour, P., Pedersen, J.S., Parts, L., Carlson, J.W., Crosby, M.A., Rasmussen, M.D., Roy, S., Deoras, A.N. *et al.* (2007) Discovery of functional elements in 12 Drosophila genomes using evolutionary signatures. *Nature*, **450**, 219-232.
5. Zhang, C., Zhang, Z., Castle, J., Sun, S., Johnson, J., Krainer, A.R. and Zhang, M.Q. (2008) Defining the regulatory network of the tissue-specific splicing factors Fox-1 and Fox-2. *Genes Dev*, **22**, 2550-2563.
6. Buckanovich, R.J. and Darnell, R.B. (1997) The neuronal RNA binding protein Nova-1 recognizes specific RNA targets in vitro and in vivo. *Mol. Cell. Biol.*, **17**, 3194-3201.
7. Jensen, K.B., Musunuru, K., Lewis, H.A., Burley, S.K. and Darnell, R.B. (2000) The tetranucleotide UCAY directs the specific recognition of RNA by the Nova K-homology 3 domain. *Proc. Natl. Acad. Sci. USA*, **97**, 5740-5745.
8. Lewis, H.A., Musunuru, K., Jensen, K.B., Edo, C., Chen, H., Darnell, R.B. and Burley, S.K. (2000) Sequence-specific RNA binding by a Nova KH domain: implications for paraneoplastic disease and the fragile X syndrome. *Cell*, **100**, 323-332.
9. Zhang, C., Frias, M.A., Mele, A., Ruggiu, M., Eom, T., Marney, C.B., Wang, H., Licatalosi, D.D., Fak, J.J. and Darnell, R.B. (2010) Integrative modeling defines the Nova splicing-regulatory network and its combinatorial controls. *Science*, **329**, 439-443.
10. Hiller, M., Zhang, Z., Backofen, R. and Stamm, S. (2007) Pre-mRNA secondary structures influence exon recognition. *PLoS Genet*, **3**, e204.
11. Shepard, P.J. and Hertel, K.J. (2008) Conserved RNA secondary structures promote alternative splicing. *RNA*, **14**, 1463-1469.
12. Bernhart, S.H., Hofacker, I.L. and Stadler, P.F. (2006) Local RNA base pairing probabilities in large sequences. *Bioinformatics*, **22**, 614-615.
13. Rabiner, L.R. (1990) A tutorial on hidden markov models and selected applications in speech recognition. *Proc. IEEE*, **77**, 257-286.

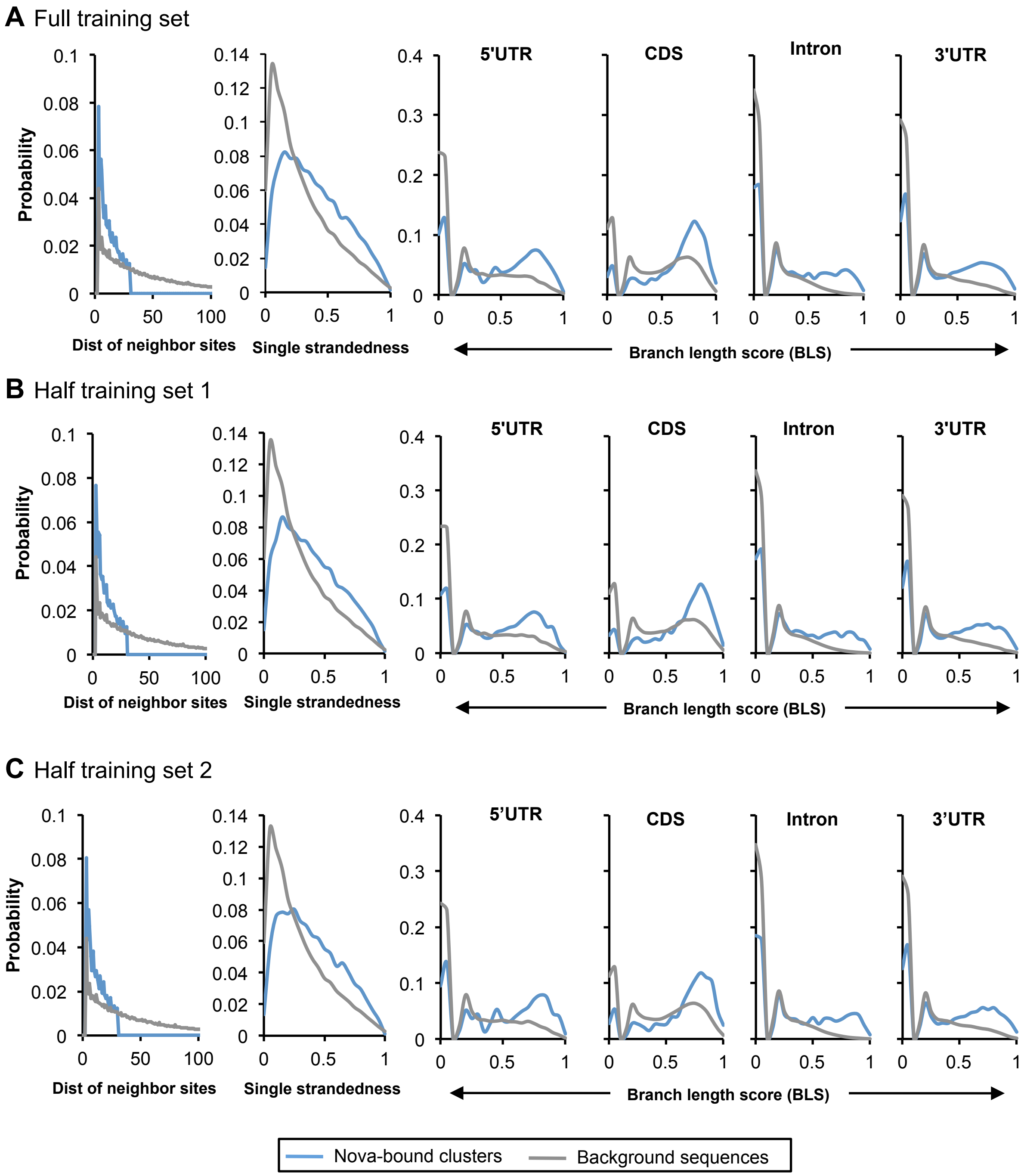

Fig. S1: (related to Fig. 1B in the main text) Emission probability distributions of spacing of Nova-binding motif sites, their accessibility and conservation.
A. Distributions estimated from the full training set, which is the same as shown in Fig. 1B in the main text. **B**,**C**. The whole transcriptome was split into two halves, and training data in each half was used to estimate the distributions. See Fig. 1B legends in the main text for more details.

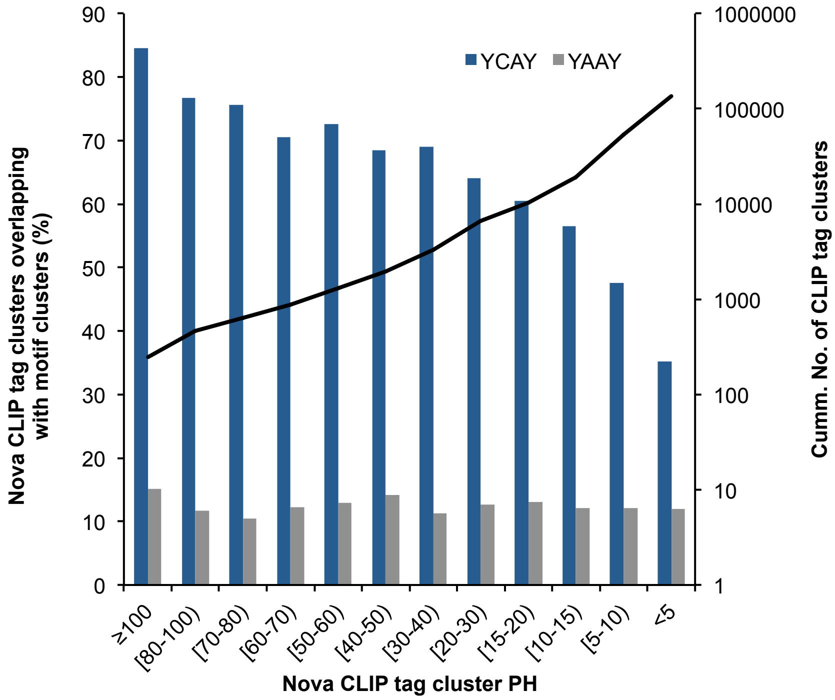

Fig. S2: The overlap between predicted YCAY clusters and CLIP tag clusters with varying peak heights (PH).

Non-repetitive CLIP tag clusters are binned according to PH and compared to YCAY clusters. For each bin, the proportion of CLIP tag cluster footprints (+/-50 nt around peak) that overlap with YCAY clusters (blue bars), or control YAAY clusters (gray bars), is shown on the left axis. The cumulative number of non-repetitive Nova CLIP tag clusters is shown as the black curve (right axis).

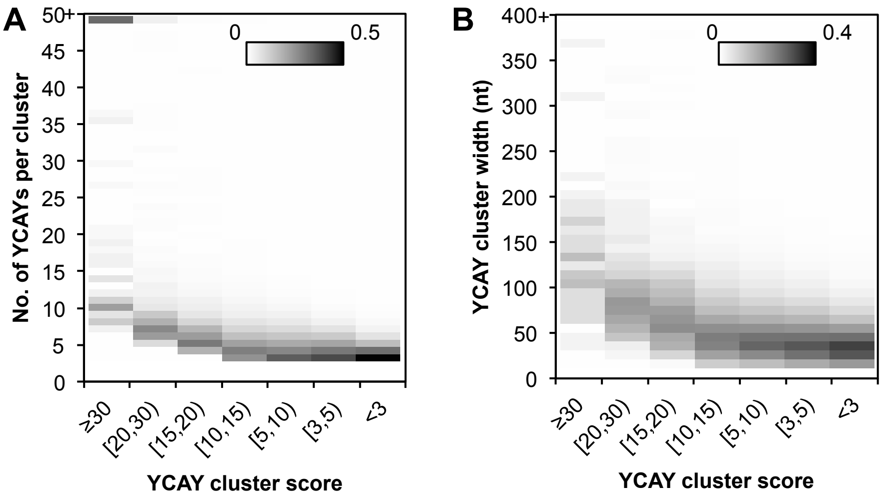

Fig. S3: General characteristics of predicted YCAY clusters.
A. YCAY clusters are divided into bins with different scores. The probability of clusters with a specific number of YCAY elements is calculated for each bin separately, and is represented in gray scale.
B. Similar to (A), but the probability of specific cluster width is shown.

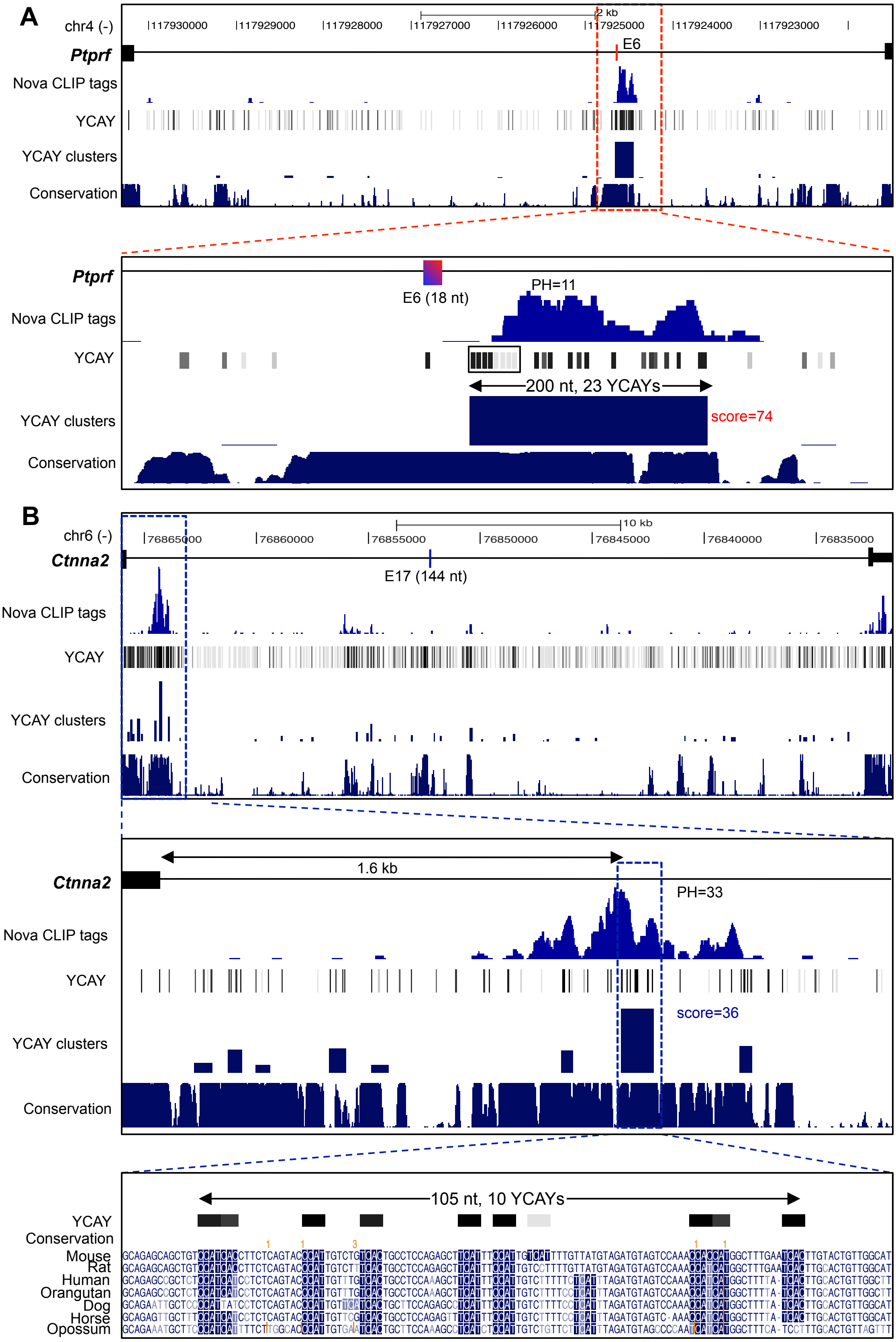

Fig. S4 (continued next page).

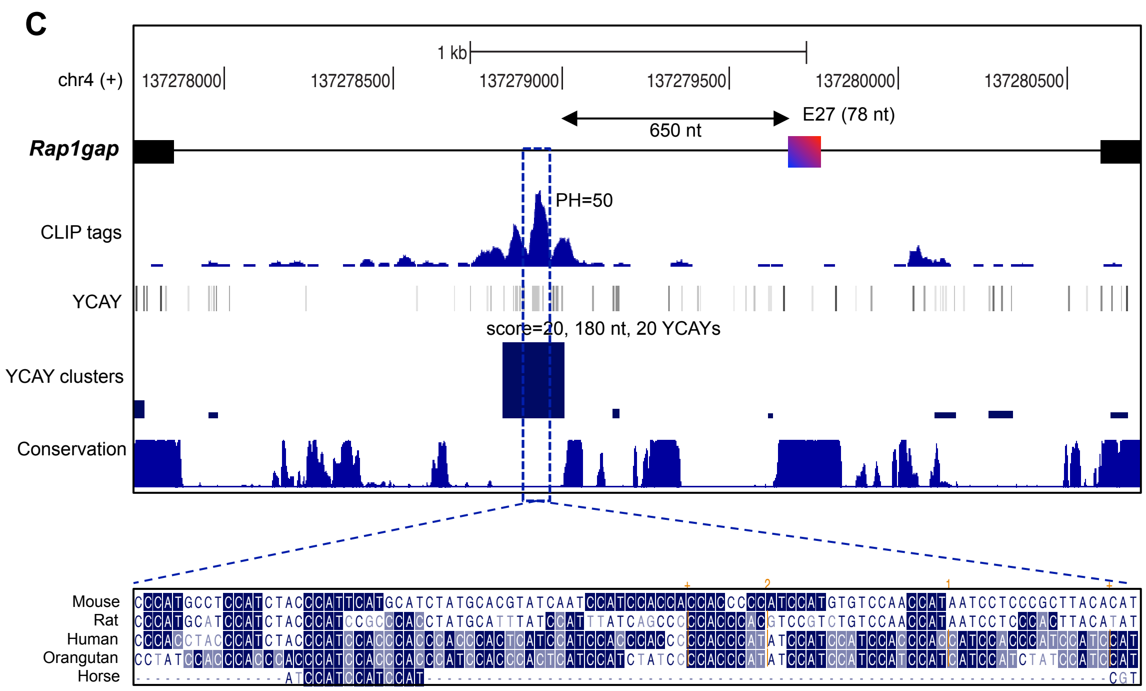

Fig. S4: Predicted Nova-bound YCAY clusters capture extensive Nova binding and distal alternative splicing regulation.

A. The predicted YCAY cluster downstream of the Nova regulated *Ptprf* exon 6 is shown. Top panel: Below the coordinates and schematic representation of the gene structure are four tracks: Nova CLIP tags, coordinates of YCAY elements with conservation (BLS) shown in gray scale, predicted YCAY clusters and 20-way mammalian phastCons scores. Bottom panel: A zoom-in view of the region flanking exon 6. The predicted YCAY cluster consists of 23 YCAY elements in a 200-nt region, and it ranks among the top of all predictions (YCAY cluster score=74). The YCAY cluster predicted by our previous analysis (Ule et al. 2006 *Nature*, 444:580-586) was indicated by a solid box in the YCAY track.

B. The predicted YCAY cluster upstream of the Nova-regulated *Ctnna2* exon 17. Top panel: Gene coordinates and structure, CLIP tags, YCAY elements and predicted clusters, and 20-way mammalian conservation are shown as in (**A**). Middle panel: a zoom-in view of the region between the upstream exon 16 and the predicted YCAY cluster, which is 1.6 kb away from the 5´ splice site. Bottom panel: A detailed view of sequences of the predicted YCAY clusters in the mouse genome and six other representative mammalian species. YCAY elements are highlighted by inversed colors. Turnover (creation and loss) of YCAY elements in the predicted cluster is tolerated and weighted by the HMM.

C. Exon 27 of *Rap1gap* is repressed by Nova. Top panel: The highest-scoring YCAY cluster (score=20), overlapping with a robust Nova CLIP tag cluster (PH=20), is located in the upstream intron, 650 nt away from the 3' splice site. This YCAY cluster consists of 20 YCAY elements, spanning 180 nt. Bottom panel: A zoom-in view of part of the predicted YCAY cluster. Extensive turnover of YCAY element (highlighted with inverted colors) in different species is observed.

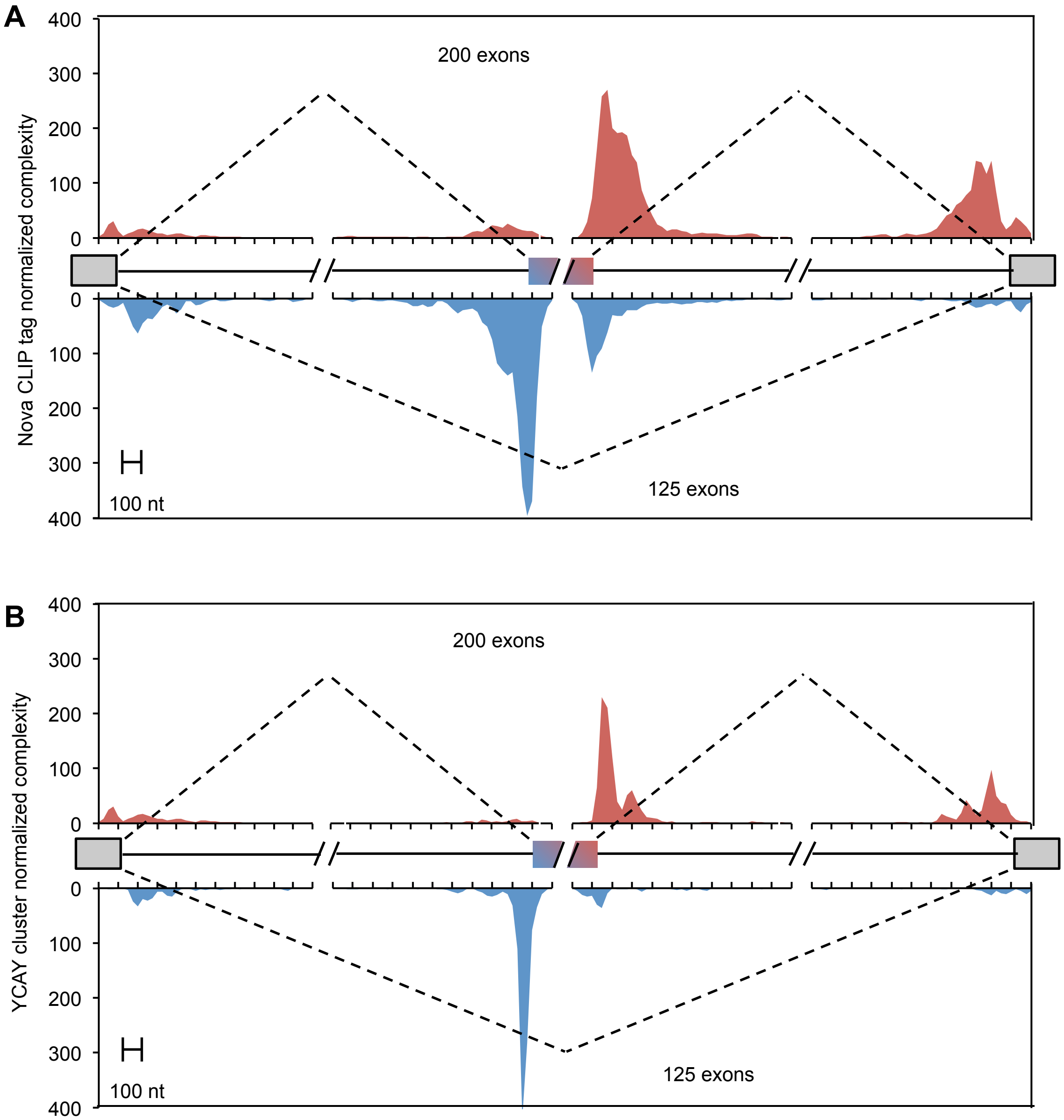

Fig. S5: The RNA map of Nova regulated alternative splicing.
A set of 325 non-redundant cassette exons regulated by Nova are shown.
A. The normalized complexity map of Nova CLIP tags for cassette exons activated (red) or repressed (blue) by Nova is shown. The number of exons in each group is indicated.
B. The normalized complexity map of YCAY clusters is displayed similar to panel (**A**).

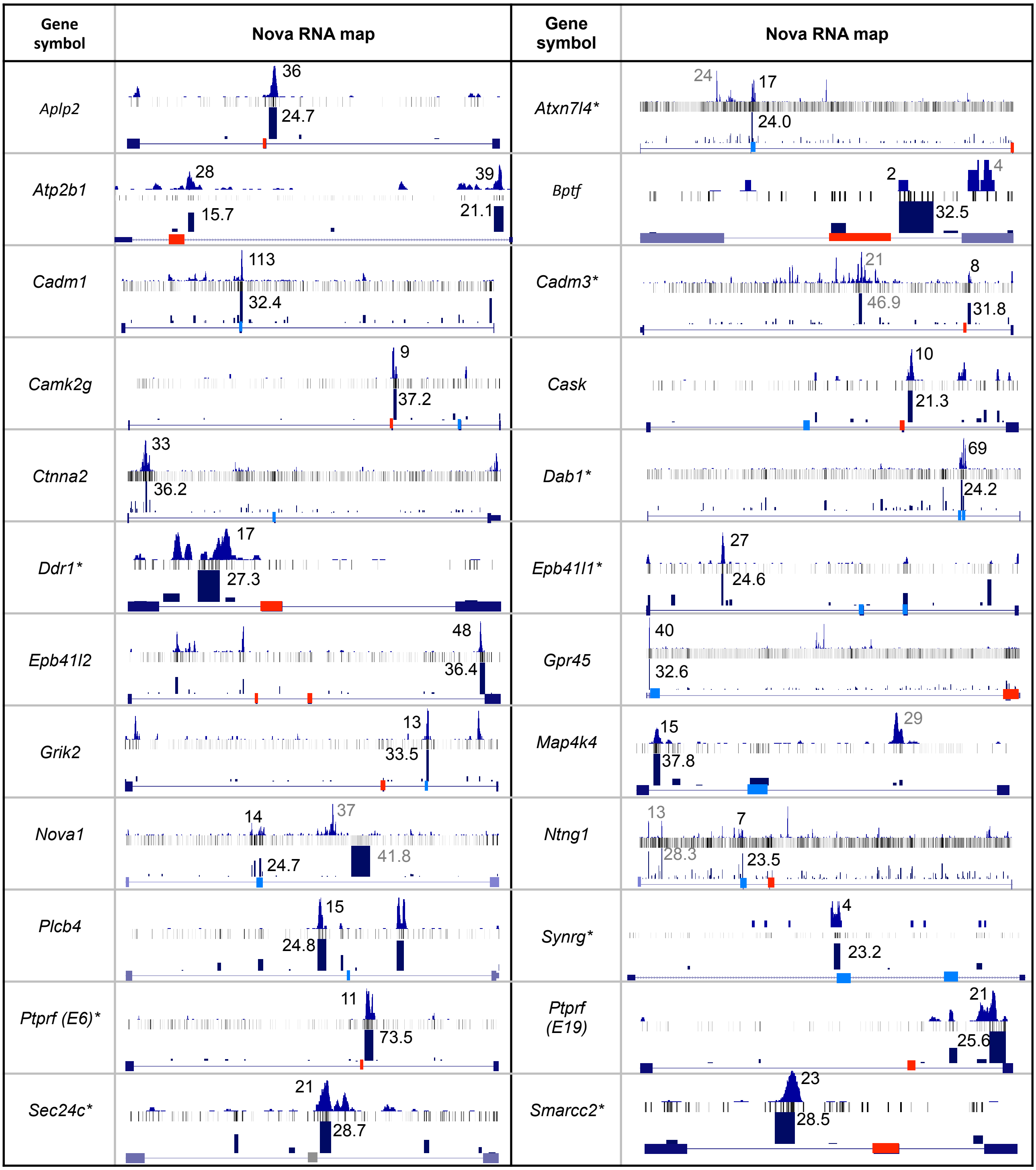

Fig. S6 legend (next page).

Fig. S6: Predicting Nova regulated alternative exons using YCAY clusters.
Exons analyzed in ref. (Ule et al. 2006 *Nature*, 444:580-586) and predicted based on mCarts YCAY cluster score (≥21.1) are shown. In each panel, the three tracks from top to bottom are distribution of CLIP tags, mCarts YCAY cluster scores, and gene structure in the alternatively spliced region. The highest-scoring YCAY clusters and major CLIP tag cluster peaks are indicated. Alternative exons with Nova-dependent inclusion and exclusion are shown in red, and blue, respectively. Exons above the "net score" threshold (≥2.7) (Ule et al. 2006 *Nature*, 444:580-586) are indicated by asterisk.

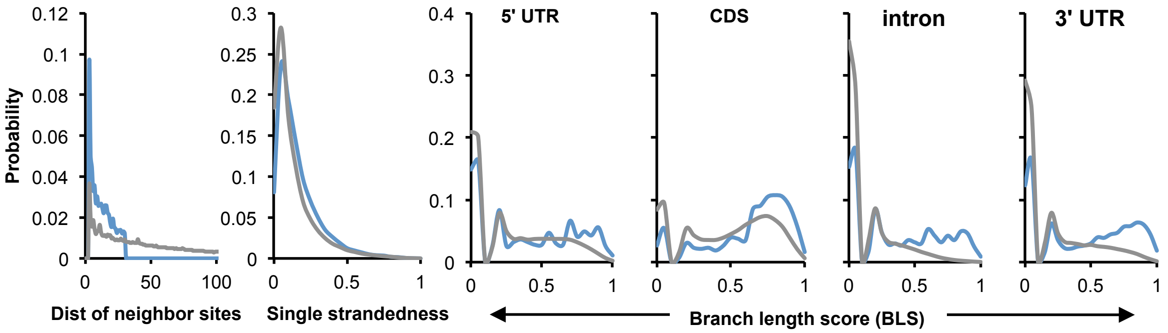

Fig. S7: Emission probability distributions of spacing of Mbnl-binding motif sites, their accessibility and conservation.
See Fig. 1B legend in the main text for more details.

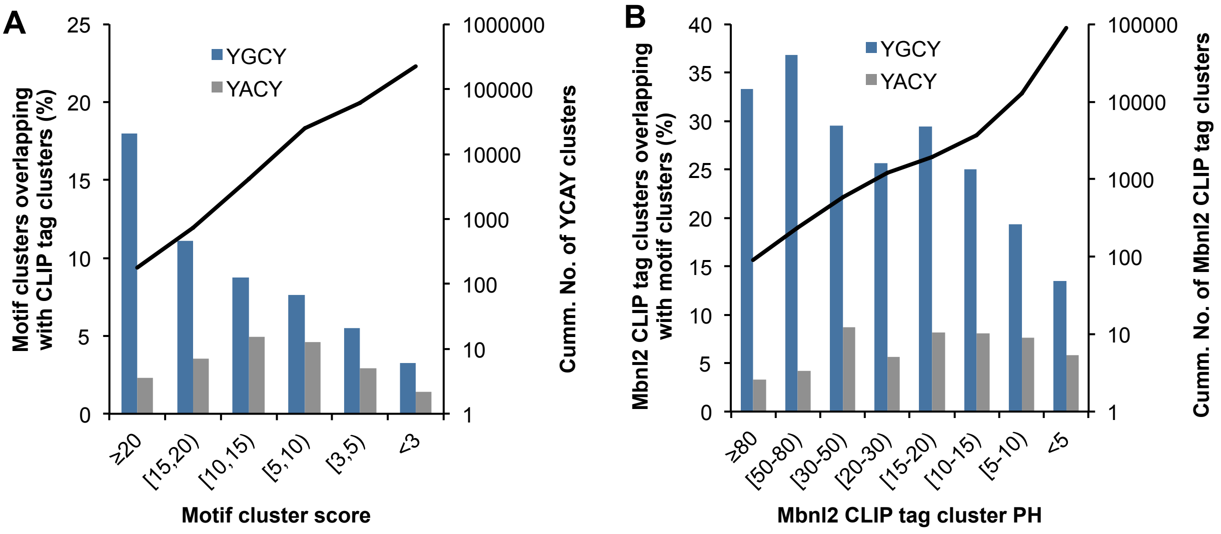

Fig. S8: The overlap between predicted YGCY clusters and Mbnl2 CLIP tag clusters depending on the stringency of each dataset.

A. The overlap between the footprints of Mbnl2 CLIP tag clusters and predicted YGCY clusters with varying scores. Non-repetitive YGCY clusters are binned into groups according to their scores. For each bin, the proportion of YGCY clusters overlapping with CLIP tag cluster footprints (+/-50 nt around peak) is shown (blue bars, left axis). YACY clusters predicted by the same model are shown (gray bars, left axis) as a control. The cumulative number of non-repetitive YGCY clusters is shown as the black curve (right axis).

B. The overlap between predicted YGCY clusters and Mbnl2 CLIP tag clusters with varying peak heights (PH). Non-repetitive CLIP tag clusters are binned according to PH and compared to YGCY clusters. For each bin, the proportion of CLIP tag cluster footprints that overlap with YGCY clusters (blue bars), or control YACY clusters (gray bars), is shown on the left axis. The cumulative number of non-repetitive Mbnl2 CLIP tag clusters is shown as the black curve (right axis).

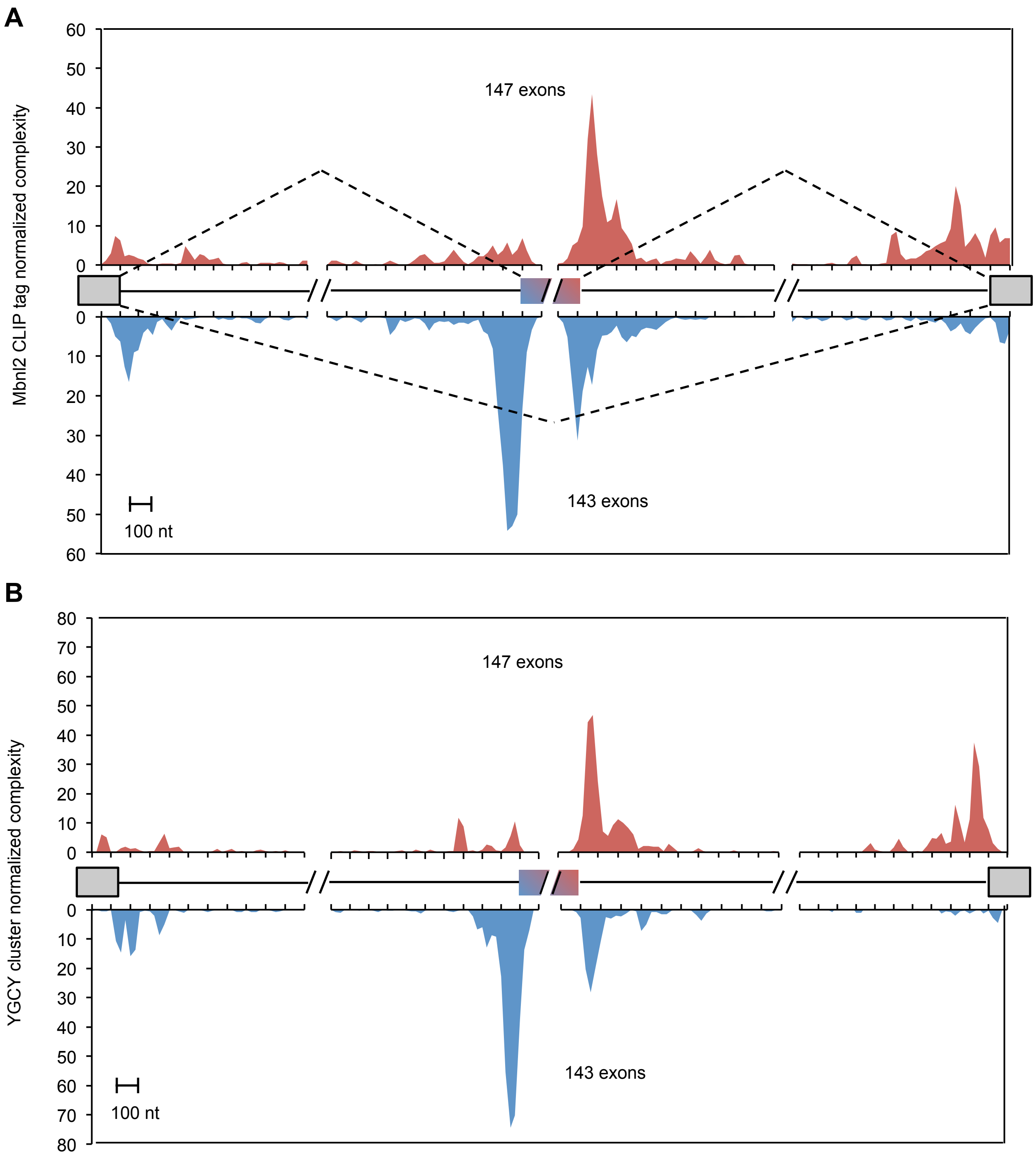

Fig. S9: The normalized complexity map of Mbnl2-regulated alternative splicing. See Fig. S5 legend for more details.

A

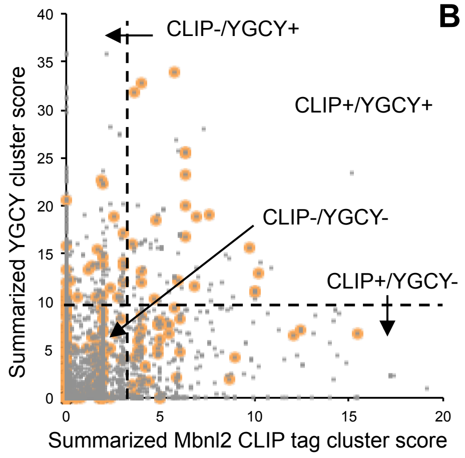

B

	YGCY+	YGCY-	Sub total
CLIP+	19/70 (27.1%)	30/325 (9.2%)	49/395 (12.4%)
CLIP-	29/322 (9.0%)	212/12,640 (1.7%)	241/12,962 1.8%
Sub total	48/392 (12.2%)	242/12,965 (1.9%)	290/13,357 (2.2%)

Fig. S10: Mbnl2-regulated alternative exons predicted from CLIP data and those predicted from YGCY clusters are complementary to each other.

A. Target exon scores predicted from CLIP data (x-axis) are plot against scores predicted from YGCY clusters (y-axis). Each dot is an exon. All cassette exons are shown in gray, and exons with Mbnl2-dependent splicing as determined by Affymetrix exon-junction microarray or RNA-Seq data are overlaid in orange. An arbitrary threshold of summarized CLIP tag cluster score (3.8) or YGCY cluster score (10) is indicated by the dotted lines.

B. Breakdown of exons according to their summarized CLIP tag cluster score or YGCY cluster score above or below the threshold. The number (orange) and percentage of exons with Mbnl2-dependent splicing in each category are also shown.
